# Intersexual chemo-sensation in a “visually-oriented” lizard, *Anolis sagrei*

**DOI:** 10.7717/peerj.1874

**Published:** 2016-03-29

**Authors:** Simon Baeckens, Tess Driessens, Raoul Van Damme

**Affiliations:** Laboratory of Functional Morphology, Department of Biology, University of Antwerp, Wilrijk, Belgium

**Keywords:** Chemical communication, Dactyloidae, Dewlap extensions, Display behaviour, Iguania, Semiochemicals, Signalling, Squamata, Tongue-flick

## Abstract

While the conspicuous visual displays of anoles have been studied in great depth, the possibility that these lizards may also interact through chemical signalling has received hardly any consideration. In this study, we observed the behaviour of male brown anoles (*Anolis sagrei*) when introduced into an environment previously inhabited by female conspecifics, and compared it to when they were introduced into an untreated environment. The males in our tests exhibited significantly more elaborate display behaviour (i.e., greater number of dewlap extensions and head-nods) and a significantly greater number of tongue extrusions while in the cage formerly occupied by females than when placed in the untreated, control cage. The absolute numbers of tongue extrusions, however, were relatively low in comparison to average tongue-flick rates of ‘true’ chemically-oriented lizards. Our results strongly suggest that the males were capable of detecting chemical cues left behind by the females. These observations provide the first evidence of intersexual chemo-sensation in an anole lizard.

## Introduction

The sensory modalities through which animals perceive the world vary greatly among taxa. Among squamate lizards, for instance, the ‘Iguania’ (Agamidae, Chamaeleonidae and Iguanidae *s*.*l*.) are often regarded as ‘visually-oriented,’ while the ‘Scleroglossa’ (all other families) are dubbed ‘chemically-oriented’ (Schwenk, 1993; Schwenk, 1994; Vidal & Hedges, 2009). Such partition is clearly flawed in the sense that many ‘chemically-oriented’ lizard species also have excellent eyesight (e.g., Pérez I de Lanuza & Font, 2014; Martin et al., 2015) and frequently use visual displays (e.g., Cooper et al., 2003; Font et al., 2012). Still, it has long been thought that the ‘visually-oriented’ Agamidae, Chamaeleonidae and Iguanidae have poor chemosensory abilities (Pratt, 1948; Evans, 1961; Alberts, Pratt & Phillips, 1992). This conviction accords well with the conventional view of squamate phylogenetic history, in which the tongue played a key role. It was believed that Scleroglossa developed a forked tongue and a sophisticated system for vomerolfaction once they acquired the ability to capture prey by the use of jaws (Schwenk, 1993; Schwenk, 1995). Instead, it is now said that the Iguania retained the putative ancestral conditions of lingual prey prehension, visual hunting, and a rudimentary vomeronasal chemosensory system (Vidal & Hedges, 2009).

More recently, several studies have shown that chemical cues are nonetheless important to iguanian lizards (Cooper, 2002). For instance, food odours elicit increased tongue-flick rates in *Dipsosaurus dorsalis*, *Pogona viticeps*, *Ctenosaura similis* and *Sauromalus ater* (Cooper & Alberts, 1990; Cooper, 2000; Cooper & Flowers, 2000; Cooper & Lemos-Espinal, 2001), and *Sceloporus jarrovi*, *S. occidentalis* and *Iguana iguana* use chemical cues in intraspecific communication (Bissinger & Simon, 1981; Duvall, 1979; Werner et al., 1987).

While a possible role for vomerolfaction has thus been accepted for other iguanid groups, chemoreception is generally considered deficient in members of the genus *Anolis.* Pratt (1948) considered the olfactory chamber of *Anolis* ‘poorly developed’ and ‘almost non-sensory,’ their Jacobson’s organ ‘reduced and completely non-sensory.’ Armstrong, Gamble & Goldby (1953) believed that the vomeronasal organs of *Anolis* species were ‘functional,’ but at the same time dubbed them ‘microsmatic,’ because the nasal sac, its epithelium and the vomeronasal organ are diminutive. Accordingly, Greenberg (1982) found that the lateral cortex, the main cortical target of olfactory sensation, was ‘virtually vestigial’ in *Anolis*. The accessory olfactory bulb, target of the vomeronasal organ, is also reduced and its subcortical target was deemed absent (Greenberg, 1982). Behavioural experiments on *A. carolinensis* failed to find any evidence that this species utilizes chemical information during prey selection (Curio & Mobius, 1978; Jaslow & Pallera, 1990), for assessing intraspecific opponents (Forster et al., 2005; Gravelle & Simon, 1980), or in mate choice (Orrel & Jenssen, 2002). These observations have discouraged further work on chemo-sensation in *Anolis*, and researchers have instead focussed on the prominent and elaborate visual displays exhibited by these animals (dewlap extensions, push-ups, head-nods etc.).

However, several lines of evidence suggest a possible role for chemical cues in *Anoli*s life history. First, individuals of the species do tongue-flick (Greenberg, 1985; Greenberg, 1993), and even more so in novel environments or when confronted with conspecifics of the same sex (Greenberg, 1993). Second, Gabe & Saint-Girons (1965) have described cloacal glands in the males of three *Anolis* species, which may function in the production of semiochemicals. Finally, in a recent comparative study of the sodefrin precursor-like factor (SPF) pheromone system, Janssenswillen et al. (2014) found 19 duplicates of a gene implicated in the production of pheromones in *A. carolinensis*.

**Figure 1:**
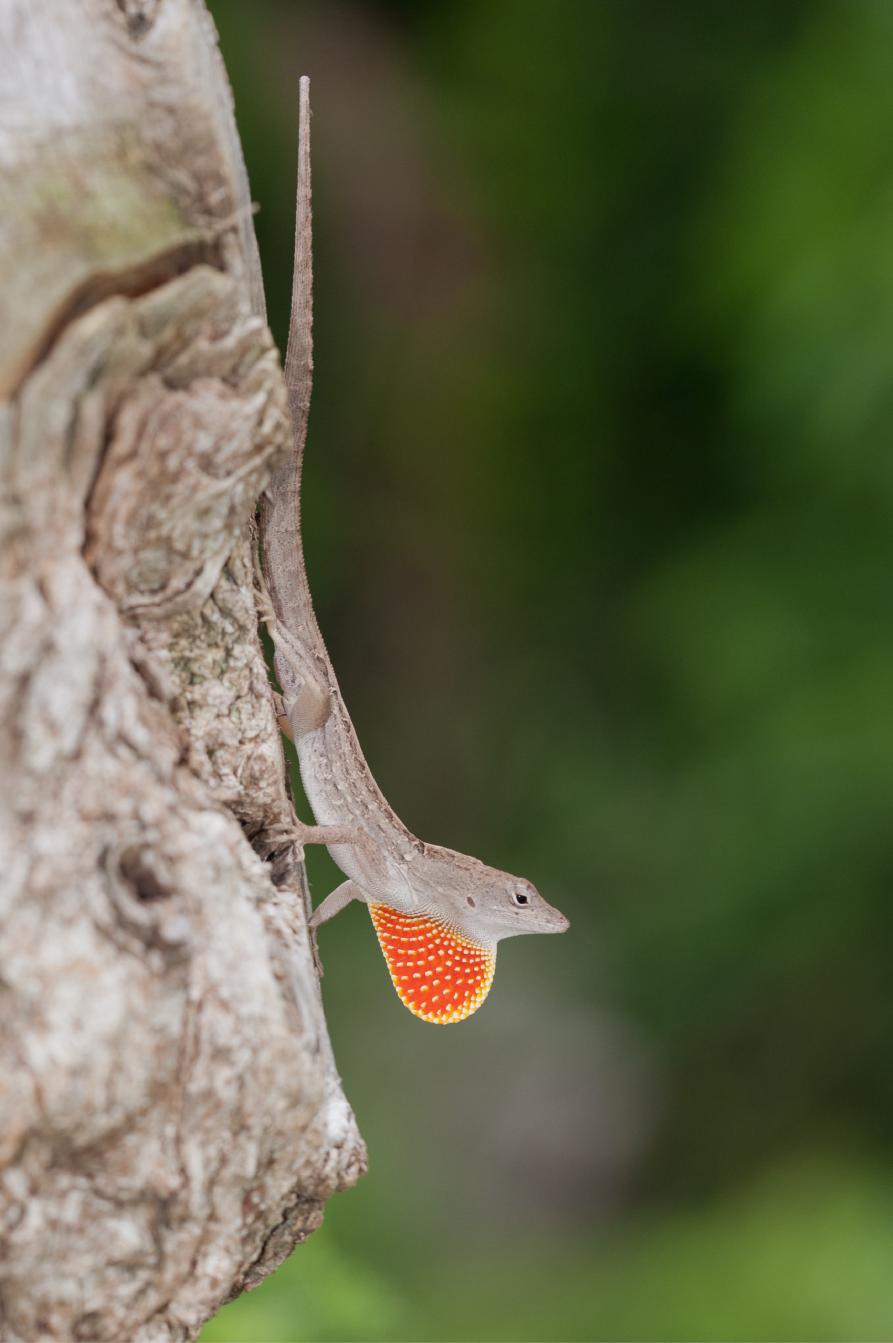
Brown anole (*Anolis sagrei*). Photograph of a male brown anole extending its dewlap. Picture taken by Steven De Decker in Santa Clara, Cuba (2012).

In this study, we observed the behaviour and tongue-flick rates in male brown anoles (*Anolis sagrei*, Fig. 1) when introduced into an environment previously inhabited by female conspecifics, and compared it to the response when in an untreated environment. Differential results provide valuable information on the intersexual chemosensory ability of *Anolis.* We predict that if male anoles are capable of detecting chemical cues left behind by the females, they will exhibit higher tongue-flick rates and more elaborate display behaviour in the experimental environment than in the control.

## Methods and Materials

### Animals and their maintenance

We purchased 14 male and four female brown anoles (*Anolis sagrei*) —which were originally caught in Florida (USA)—via the pet trade (Fantasia Reptiles, Belgium, license HK51101419). Males and females were housed separately, with a maximum of four individuals per terrarium (100 × 40 × 50 cm). The female cage was isolated from the cages containing males in order to avoid any visual or chemical contact between them. Each cage contained a layer of peat bedding covered with banana-leafs and several wooden perches (length 40 cm; diameter 3 cm). A 60-watt bulb suspended above one end of the terrarium provided heat and light (12 h/d). Lizards were hand-sprayed with water every other day, had access to fresh water at all times, and were fed crickets (*Acheta domesticus*) three times a week. The lizards were housed in one room whilst the experiments took place in a separate room. All work was carried out in accordance with the University of Antwerp animal welfare standards and protocol (ECD 2011-64).

### Experimental design

Our experimental procedure consisted of introducing male anoles into two distinct unfamiliar environments: (i) a control and (ii) an experimental terrarium. Both glass terraria (50 × 50 × 50 cm) were completely closed and contained a layer of peat bedding, two identical wooden perches, and a 60-watt light bulb. We took great care to ensure that the appearance of both cages was as similar as possible. One side of the cage was coated with a dark window film (Johnson Window Films), which filters light transmission. The coating enabled us to observe the lizard in the test cage without being visually noticeable to the lizard itself. We chose this method instead of a one-way mirror, as Driessens, Vanhooydonck & Van Damme (2013) reported that a mirror could affect the behaviour of brown anole males. All other sides of the cage were covered and taped with white paper to make the terrarium non-transparent, and hence to avoid any kind of external visual stimuli. After every observation, we removed the bedding, washed the terrarium and perches with odourless detergent and afterwards with ethanol (70%), and left it to dry. The bedding was replaced between subsequent trails in order to remove any chemical stimuli left by lizards from the previous trial. All observations took place in a separate room from where the lizards were housed.

The control set-up consisted of an untreated terrarium, whereas the experimental terrarium was formerly inhabited by four female conspecifics. Prior to observation, all females were translocated from their home cage to the experimental terrarium, where they were housed for a minimum of 8 h. Females were removed from the experimental terrarium to their home cage 5 min before each test; so the male lizards were only exposed to the chemicals left by females, not to visual or auditory female stimuli. The researcher wore fresh disposable gloves whilst handling the lizards, in order to avoid contamination with human odours. Every male was exposed to the control and experimental terrarium in a randomized order, and tested only once a day. The use of terraria (control vs. experimental) was also randomized. Thus, all 14 males were observed twice: once in the control terrarium and once in the experimental terrarium (so, *n* = 28).

All experiments were conducted in the reproductive season of *A. sagrei* (August–September 2015) and the observations were made during the lizards’ peak activity hours (10:00-16:00).

### Observations

Observations started approximately 10 s after the male lizard’s introduction into the terrarium and lasted for 20 min. The lizard’s behaviour was monitored and scored online using the software JWatcher (version 1.0; Blumstein & Daniel, 2007). Following Driessens, Vanhooydonck & Van Damme (2013) we distinguished between three visual display types: dewlap extensions, head-nods, and push-ups. A dewlap extension was defined as one complete extension and retraction of the dewlap, a head-nod as one single up and down movement that involved only the head, and a push-up as one single up and down movement of the whole body caused by flexion of only the front legs or all four legs. The number of display events was scored, and the time duration of exhibiting display behaviour was recorded. As a measure for exploratory behaviour we scored the number of tongue extrusions and total duration (in seconds) of locomotor behaviour (walking, running, jumping).

### Statistics

To examine differences in behavioural states and events we used generalized estimating equations with repeated measures. The analyses were run with “treatment” (control vs. experimental) as within-subject variable. Display events and tongue extrusion counts were assumed to follow a Poisson distribution (loglinear model type). Time duration data were transformed (square root) to ensure normality (*Shapiro—Wilk’s test* with *W* ≥ 0.95). All statistical analyses were conducted in SPSS v. 22.0 (Chicago, IL, USA) and *P* < 0.05 was considered statistically significant.

## Results

**Figure 2:**
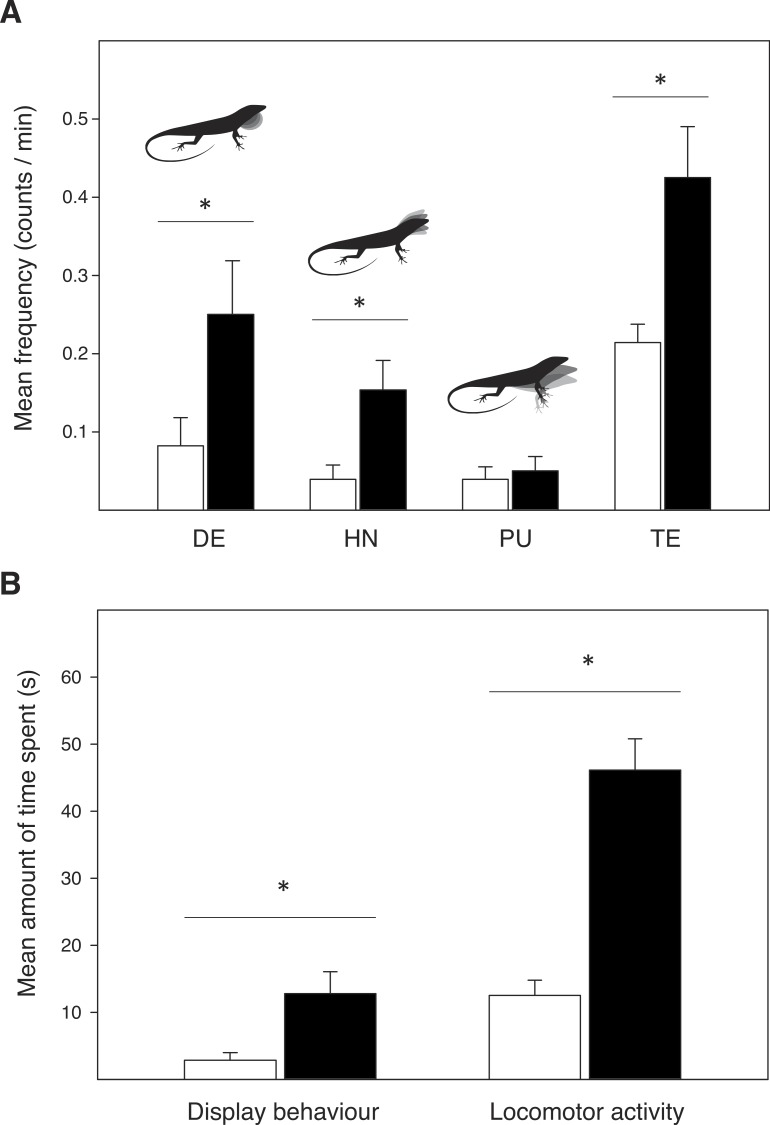
Display behaviour and exploratory activity in male *Anolis sagrei* when introduced in an untreated control terrarium (*white bars*) and an experimental terrarium previously inhabited by conspecific females (*black bars*). (A) Mean display frequency and tongue extrusion (TE) rate, as counts per minute. Display events include dewlap extensions (DE), head-nods (HN) and push-ups (PU). (B) Mean amount of time spent (in seconds) on display behaviour and locomotor activity during the 20 min observation trials. Error bars represent SE, and asterisks represent significant differences: ^∗^*P* < 0.01.

Male *A. sagrei* showed significantly more dewlap extensions (*Wald χ*^2^ = 4.817, *P* = 0.028) and head-nods (*Wald χ*^2^ = 7.026, *P* = 0.008) in the experimental female cage, than in the untreated control cage (Fig. 2A). No difference was found in the number of push-ups (*Wald χ*^2^ = 0.370, *P* = 0.543). The average amount of time spent displaying was highest in the experimental cage (*Wald χ*^2^ = 10.770, *P* = 0.001; Fig. 2B). Males also extruded their tongue more often (*Wald χ*^2^ = 13.440, *P* < 0.001) and showed more active locomotor behaviour (i.e., sum of the total time walking, running and jumping: *Wald χ*^2^ = 69.477, *P* < 0.001) in the experimental cage compared to the control cage.

## Discussion

In our tests, male *Anolis sagrei* lizards exhibited more display and exploratory behaviour when introduced into a novel environment previously inhabited by female conspecifics, than when placed in a novel, untreated cage. Our results strongly suggest that the males were capable of picking up chemical cues left by the females. Our observations constitute the first evidence of intersexual chemo-sensation in an anole lizard.

One of the reasons why chemical communication has been understudied in *Anolis*, is that anoles do not possess epidermal glands (Mayerl, Baeckens & Van Damme, 2015). Epidermal gland secretions are generally considered the main source of chemicals involved in lizard chemical communication (Martín & López, 2014; Martín & López, 2015). However, such glands are also lacking in other lizard groups, such as anguids and skinks that nevertheless use semiochemical cues. In these groups, semiochemicals are produced in the cloaca or the integument (Duvall, Herskowitz & Trupiano-Duvall, 1980; Gonzalo et al., 2004; Head et al., 2008; Scott et al., 2015). For example, lipid fractions of the urodeal glands of female broad-headed skink (*Plestiodon laticeps*) elicit courtship behaviour in conspecific males (Cooper & Garstka, 1987), and cloacal glands of both sexes produce species-specific semiochemicals (Cooper, Garstka & Vitt, 1986; Cooper & Vitt, 1987; Trauth et al., 1987). Also, male *P. laticeps* are able to discriminate between sexes, based on skin chemicals alone (Cooper & Vitt, 1984a; Cooper & Vitt, 1984b). Cloacal glands have been described in males of *Anolis cristatellus, A. evermanni* and *A. pulchellus* (Gabe & Saint-Girons, 1965). Unfortunately, no information is available on the presence/absence of cloacal glands in sexually active female anoles (D Sever & D Siegel, pers. comm., 2015). Still, it is highly likely that while moving around, females passively deposited chemicals of various origins (cloacal/urodeal secretion, faecal excrements, skin fragments) on the substrate, which were later perceived by male (vomer)olfaction.

Another possible explanation for the dearth of studies on *Anolis* chemo-sensation is their patent use of visual displays. The anole dewlap has become a model system in communication biology (Jenssen, 1977; Carpenter, 1978; Nicholson, Harmon & Losos, 2007). The attractiveness of the dewlap as a visual signalling device may have diverted attention away from other sensory channels. Admittedly, although *A. sagrei* males in the cages labelled with female odours displayed significantly more than males in untreated cages, the absolute numbers of displays shown were low compared to those exhibited by males in visual contact with females. Driessens, Vanhooydonck & Van Damme (2013) observed male *A. sagrei* in captive conditions similar to ours but in visual contact with female conspecifics, and reported dewlap extension rates nearly nine times higher than those recorded in this study (counts per minute, cpm: $\bar {x}=0.25$, SE = 0.07, *n* = 14 vs. $\bar {x}=2.19$, SE = 0.35, *n* = 27). Possibly, males in our experiments were awaiting visual confirmation of the chemical signals before engaging in full visual signalling displays (which may be costly, Leal, 1999; Simon, 2007; Lailvaux, Gilbert & Edwards, 2012).

A third observation that plausibly discouraged former researchers to study chemosignalling in *Anolis*, is their low baseline rate of tongue extrusions. In our tests, male anoles extruded their tongue on average 0.21 ± 0.02 cpm in the control cage. Comparing this rate to tongue-flick rates of ‘true’ chemical-oriented lizards, demonstrates the low tongue-flick rate in anoles all the more (Table 1). Verwaijen & Van Damme (2007) observed relative high tongue-flick rates in several lacertid species, such as *Podarcis muralis* (4.60 ± 0.50 cpm), *Psammodromus algirus* (4.20 ± 0.60 cpm), *Takydromus sexlineatus* (3.30 ± 0.50 cpm) and *Acanthodactylus erythrurus* (2.60 ± 0.50 cpm). Bissinger & Simon (1979) observed the vomerolfactory behaviour of lizards of different taxa, in semi-natural zoo conditions. Their results suggest that only cordylids (e.g., *Smaug warreni:* 0.19 ± 0.01 cpm) have lower average tongue-flick rates than the anoles in our study. Even in comparison to other iguanids, brown anoles score fairly low (e.g., *Ctenosaura clarki*: 0.43 ± 0.18 cpm). Regardless of anoles baseline rate, our tests do show a significant increase in tongue extrusion rate when confronted with female odours.

**Table 1:** Tongue-flick rates in lizards. Overview of baseline tongue-flick rates in lizards of various taxa, observed in semi-natural settings—reported in counts per minute (cpm). Means and standard errors (SE) are shown.

Family	Species	Baseline tongue-flick rate Mean ± SE (cpm)
Cordylidae^a^	*Smaug warreni*	0.19 ± 0.01
**Dactyloidae**^b^	***Anolis sagrei***	**0.21 ± 0.02**
Phrynosomatidae^a^	*Sceloporus jarrovi*	0.27 ± 0.12
Iguanidae^a^	*Ctenosaura clarki*	0.43 ± 0.18
Gerrhosauridae^a^	*Zonosaurus madagascariensis*	1.21 ± 0.31
Lacertidae^c^	*Acanthodactylus aureus*	2.10 ± 0.30
Lacertidae^c^	*Acanthodactylus erythrurus*	2.60 ± 0.50
Lacertidae^c^	*Takydromus sexlineatus*	3.30 ± 0.50
Lacertidae^c^	*Psammodromus hispanicus*	3.60 ± 0.50
Lacertidae^c^	*Psammodromus algirus*	4.20 ± 0.60
Lacertidae^c^	*Podarcis peloponnesiacus*	4.20 ± 0.50
Lacertidae^c^	*Zootoca vivipara*	4.30 ± 0.60
Lacertidae^c^	*Podarcis muralis*	4.60 ± 0.50
Scincidae^a^	*Tiliqua scincoides*	5.51 ± 0.96
Helodermatidae^a^	*Heloderma suspectum*	7.85 ± 0.81
Teiidae^a^	*Aspidoscelis exsanguis*	11.95 ± 1.99

**Notes.**

a Bissinger & Simon (1979).

b This study.

c Verwaijen & Van Damme (2007).

While the change in tongue extrusion rates strongly implies the use of anole vomerolfaction, we cannot rule out the use of olfaction in which (only) volatile chemicals are processed by the nasal organs (Cooper & Burghardt, 1990), as Gabe & Saint-Girons (1976) have suggested the anole olfactory epithelium to be more developed then their vomeronasal organ. Dactyloidae also possess large numbers of tongue taste buds, but the use of lingual gustation in squamate chemosensory discrimination is said to be ‘inadequate’ (Cooper, 1997). Although for the human eye, visual hints of the former presence of females were not present, we cannot rule out the possibility that males perceived the females visually, rather than chemically. For instance, it is known that the femoral gland secretion of the iguanid *Dipsosaurus dorsalis* strongly absorb ultraviolet light, which enables these femoral deposits to act as visual markers for locating low volatility semiochemicals at far range (Alberts, 1989).

The observation that males respond towards female chemical cues implies signalling multimodality in *Anolis sagrei*. Moreover, the fact that both chemical and visual stimuli (Driessens, Vanhooydonck & Van Damme, 2013; Driessens et al., 2015) elicit similar behaviour in male conspecifics, suggest that both signalling channels broadcast analogous information. Repeating the same message in different ways can enhance or ensure information transmission (Johnstone, 1996; Rowe, 1999).

In summary, our study provides the first reported evidence of intersexual-induced chemo-sensation in anoles. The chemical cues of these lizards endeavours to persist in absence of the signaller, providing information on the former presence of conspecifics in a given environment.

## Supplemental Information

10.7717/peerj.1874/supp-1 Data S1 Table with descriptive statistics for the behavioural traits scored Descriptive statistics for the display behaviour and exploratory activity in male *Anolis sagrei* when introduced in an untreated control terrarium (‘control’) and an experimental terrarium previously inhabited by conspecific females (‘exper’).

## Additional Information and Declarations

### Data Availability

The following information was supplied regarding data availability: The raw data has been supplied as a Data S1.
