# Supplementary material for: Intersexual chemo-sensation in a “visually-oriented” lizard, Anolis sagrei
Source: PeerJ. 2016 Mar 29;4:e1874. doi: 10.7717/peerj.1874 (PMC4824904; doi:10.7717/peerj.1874)
Supplement: Data S1 — Descriptive statistics for the display behaviour and exploratory activity in male Anolis sagrei when introduced in an untreated control terrarium (‘control’) and an experimental terrarium previously inhabited by conspecific females (‘exper’). [file peerj-04-1874-s001.pdf]

Table with descriptive statistics for the behavioural traits scored — Descriptive statistics for the display behaviour and exploratory activity in male *Anolis sagrei* when introduced in an untreated control terrarium ('control') and an experimental terrarium previously inhabited by conspecific females ('exper').

	**Behavioural trait**	**average**	**SE**	***n***
frequency (counts / min)	DE (control)	0.082	0.036	14
	DE (exper)	0.250	0.069	14
	HN (control)	0.039	0.018	14
	HN (exper)	0.154	0.038	14
	PU (control)	0.039	0.016	14
	PU (exper)	0.050	0.018	14
	TE (control)	0.214	0.023	14
	TE (exper)	0.425	0.065	14
mean amount of time spent (seconds)	Display beh. (control)	2.831	1.164	14
	Display beh. (exper)	12.742	3.305	14
	Locomotion beh. (control)	12.530	2.214	14
	Locomotion beh. (exper)	46.113	4.680	14

DE, dewlap extension; HN, head-nod; PU, push-up; TE, tongue extrusion; SE, standard error
